# Assessing Internalizing Symptoms and Their Relation with Levels of Impairment: Evidence-Based Cutoffs for Interpreting Inventory of Depression and Anxiety Symptoms (IDAS-II) Scores

**DOI:** 10.1007/s10862-022-10008-6

**Published:** 2023-01-10

**Authors:** A. De la Rosa-Cáceres, O. M. Lozano, M. Sanchez-Garcia, F. Fernandez-Calderon, G. Rossi, C. Diaz-Batanero

**Affiliations:** 1 Department of Clinical and Experimental Psychology, Facultad de Ciencias de La Educación, University of Huelva, 21071 Huelva, Spain; 2 Research Center for Natural Resources, Health and the Environment, University of Huelva, Huelva, Spain; 3 Personality and Psychopathology Research Group (PEPS), Department of Psychology (PE), Vrije Universiteit Brussel (VUB), Brussels, Belgium

**Keywords:** Depression, Anxiety, Emotional Disorders, Impairment, Assessment, PROMs, Cutoffs

## Abstract

Tests and scales measuring psychological disorders should provide information about how scores relate to other constructs such as quality of life or functional impairment. Such information is necessary to allow that their scores contribute to clinical decision making. The current study analyzes the clinical utility of the Spanish version of the Inventory for Depression and Anxiety Symptoms (IDAS-II) to discriminate between different levels of functional impairment and identify the IDAS-II scales that contribute most to explaining impairment. The total sample (*N* = 1390) consists of two subsamples: a community sample of the general population (*n* = 1072) selected by random sampling; and a sample of patients (*n* = 318) from public and private mental health services. The Spanish IDAS-II for measuring internalizing symptoms and WHODAS 2.0 for measuring impairment were administered to all participants. All scales show statistically significant higher scores in the patient sample, with Cohen's *d* effect sizes values greater than 0.30, except for well-being (*d* = 0.19). The cutoff values and their confidence intervals do not overlap with the means of either the community or patient sample. AUC values for most of the scales are above .70, except for appetite gain, ordering, euphoria, cleaning, and well-being. Multiple linear regression model using IDAS-II scales explain 57.1% of the variance of the WHODAS 2.0 (*F*_12.1377_ = 155.305; *p* < .001). Cutoff values provided allow us to reliably differentiate between the patients and community samples. Spanish IDAS-II scores show greater sensitivity and specificity in detecting those with greater impairment. General Depression, Lassitude, Panic and Claustrophobia contribute to impairment in a greater extent. Knowledge of which symptoms are most related with impairment, allows healthcare providers to improve treatment planning based on empirical evidence.

## Introduction

Mental disorders are among the leading causes of years lived with disability worldwide and a major cause of health burden (Global Burden of Diseases – GBD Mental Disorders Collaborators, 2022). Among them, depression and anxiety disorders contributed most to the proportion of mental disorder disability-adjusted life years in 2019 (37.3% and 22.9%, respectively) (Whiteford et al., 2013). Further, as a consequence of the impact of COVID-19, the prevalence of these disorders remained high or even increased (Kumar & Nayar, 2020), with studies indicating rates in the general population of up to 34.31% and 38.12% for depression and anxiety, respectively (Necho et al., 2021). These emotional disorders can present with different degrees of severity, ranging from mild to severe and with different symptomatology. More severe symptomatology is generally associated with significant impairment (Hasin et al., 2018; Hammer-Helimich et al., 2018; Morin et al., 2020), requiring specialized attention from psychiatrists and psychologists who will evaluate the therapeutic needs.

The diagnosis of presence and severity of these disorders has generally been made following the Diagnostic and Statistical Manual of Mental Disorders (DSM) and International Classification of Diseases (ICD) classification systems. Tests based on these nosotaxies determine the severity and absence/presence of these mental disorders, based on a count of diagnostic criteria and a categorical cutoff for disorder presence. These tests are thus essential for diagnosing and monitoring patients (Jablensky, 2016), although some authors question basing clinical decisions on the count of diagnostic criteria (Lane & Sher, 2015; Markon et al., 2011; Østergaard et al., 2011; Zimmerman et al., 2015).

As a complement to the above instruments, other scales and tests assessing emotional disorders use dimensional approaches, that ease the correspondence between scale and test scores and indicators of severity (Krueger et al., 2018; Stanton et al., 2020) and allow to infer the severity of disorders by comparing to test norms (i.e. standard of percentile norms). For example, scales such as the Depression Anxiety Stress Scales (DASS; Crawford & Henry, 2003), the Hospital Anxiety and Depression Scale (Crawford et al., 2001; Hinz & Brähler, 2011) or the Inventory of Depression and Anxiety Symptoms (IDAS-II; Nelson et al., 2018; Sánchez-García et al., 2021) have available reports of the score percentiles obtained in random samples extracted from general population. These allow clinicians to infer the severity of a patient’s disorder locating his or her score on the patient’s reference group percentiles (i.e. norms). Also, these instruments give the opportunity to detect greater heterogeneity between individuals (compared to categorical diagnostic approaches) and are thus more sensitive to measurement of patients’ change during the therapeutic process (Kraemer et al., 2004).

The interpretation of scores based on diagnostic criteria count and normative cutoffs are complementary approaches, and both provide useful information that enrich clinical judgment (Trivedi, 2009). However, several authors argue that tests and scales measuring psychological disorders should also provide information about how scores relate to other constructs such as quality of life or functional impairment (Fried et al., 2022; McKnight & Kashdan, 2009). This is especially important in the measurement of depression and anxiety, due to the strong influence of these disorders on impairment and social daily life (Rapaport et al., 2005). Symptoms most strongly related to functional impairment in psychiatric patients are fatigue, concentration problems and negative alterations in mood (Tanner et al., 2019). Previous evidence suggests that symptoms most differentiating clinical and non-clinical samples include lassitude (related with fatigue) and dysphoria for depression (Stasik-O’Brien et al., 2019; Watson et al., 2012; Watson & O’Hara, 2017) or panic attacks and claustrophobia in the case of anxiety symptoms (Irak & Albayrak, 2020). In the case of depression, it is reported that only 41.9% of patients respond to treatment, indicating that most of patients still have substantial functional impairment even after treatment (McKnight & Kashdan, 2009). Concerning anxiety disorders, reductions in symptom severity and restoration of function, while related, appear to be disorder-specific (McKnight et al., 2016). Therefore, disentangling which depression and anxiety symptoms are most related with functioning and providing guiding lines for clinicians is a priority.

In this sense, patient-report outcomes measures (PROM) are crucial methods to evaluate the impact of mental disorders/physical illnesses and their treatment on daily life (Lloyd et al., 2014; Yorkston & Baylor, 2019). These instruments are generally easy to administer and have proven useful for monitoring patients in mental health services (Knaup et al., 2009; Øvretveit, et al., 2017; Shimokawa et al., 2010). For example, clinician experts in insurance medicine might take the empirical evidence for PROMs into account in the decision making for recommending medical leaves or disability benefits (Tanner et al, 2019). Among the PROMs, both the DSM-5 (American Psychiatric Association, 2013) and some authors (Obbarius et al., 2017) highlight the usefulness of the WHODAS 2.0 (World Health Organization, 2000). The WHODAS 2.0 is an instrument that differentiates between different levels of impairment according to the International Classification of Functioning, Disability and Health (ICF). In addition, the WHODAS is adapted to numerous languages and has shown adequate psychometric properties in diverse populations (e.g. Ćwirlej-Sozańska et al., 2020; Federici et al., 2022; Koumpouros et al., 2018; Saltychev et al., 2021).

However, evidence from daily clinical practice reflects that use of PROM is implemented by less than 20% of clinicians (Lewis et al., 2020), despite the existing recommendations (Knaup et al., 2009; Shimokawa et al., 2010). The lack of clinically meaningful information or the effort required to administer these tests might hinder regularly use in clinical sessions (Campbell et al., 2021; Gelkopf et al., 2022). In addition, for tests and scales to be routinely administered in clinical sessions, it is necessary that their scores contribute to clinical decision making, such as drug administration, hospitalization, sick leave, etc. (Kraemer et al., 2004; Sharma, 2021; Widiger & Samuel, 2005). Therefore, it might be useful to provide clinicians information on the impairment of patients through the scales that assess mental disorders such as anxiety and depression, given these scales are more frequently used than PROM instruments. Research studies are consequently needed that establish associations between the scores of tests measuring mental disorders and the ICF levels. Thus, and consistent with the Consensus-based Standards for the selection of health Measurement Instruments (COSMIN; Mokkink et al., 2016), test scores assessing symptoms of mental disorders such as anxiety and depression would also allow a qualitative interpretation in terms of impairment.

Bearing in mind the above, the general objective of the current study is to analyze the clinical utility of the Spanish version of IDAS-II (De la Rosa-Cáceres et al., 2020), an instrument that assesses internalizing symptoms (including anxiety and depression symptoms), by evaluating its ability to discriminate between different levels of functional impairment (as measured by the WHODAS 2.0). Given more impairment can be expected in treatment-seeking samples (Irak & Albayrak, 2020; Watson et al., 2012; Watson & O’Hara, 2017), the Spanish version of IDAS-II should also be able in this study to discriminate patient from community samples in terms of scores obtained (with patient samples having higher mean scores compared to community samples in analogy to earlier studies). In addition, this study examines which emotional disorders symptoms contribute most to explain the total degree of impairment being found. In order to address the general objective, three specific objectives have been established: 1) examine the ability of the Spanish version of IDAS-II (De la Rosa-Cáceres et al., 2020) to discriminate between community and patient samples and providing clinical cutoffs for each IDAS-II scale; 2) identify the cut-off for the Spanish IDAS-II scales associated with moderate and severe impairment according to the ICF on the whole sample (as measured with the WHODAS 2.0); 3) identify the Spanish IDAS-II scales that contribute most to explaining impairment, as indexed by the WHODAS 2.0 total scale score.

According to previous research, it is hypothesized that scores in the clinical sample will be higher than those in the non-treatment seeking community sample, especially on the General depression and Dysphoria scales, which will present the largest effect sizes (Irak & Albayrak, 2020; Watson & O'Hara, 2017). Cutoffs for moderate and severe impairment levels have not been determined by previous studies, yet are expected to be discriminative (AUC values > .7) for all scales except for Well-Being, as higher well-being scores should not be associated with greater impairment. Finally, similar to Tanner et al. (2019), the scales most associated with functional impairment are expected to be Lassitude, General depression and Social anxiety.

## Methods

### Participants

The total sample (*N* = 1390) consists of two subsamples: a community sample of the general population (*n* = 1072) and a sample of patients (*n* = 318). The 1,072 participants in the community sample were selected by random sampling, divided into strata representative of the Spanish population for gender, age and geographical region of Spain. The patient sample consisted of 318 patients from public and private mental health services in the province of Huelva (Spain). Inclusion criteria for both samples (community and patients) were as follows: 1) be at least 18 years of age; 2) sign the informed consent; 3) not have any medical or psychological diagnosis that would preclude the administration of the tests. The clinical sample also met the following inclusion criteria: 1) being under treatment in a mental health service during the data collection; 2) have been diagnosed with a mental disorder according to DSM diagnostic criteria (at time of data collection, DSM-IV was used in clinical practice). These diagnoses were only used to determine whether patients were eligible for the clinical group, the specific diagnoses were not used in the analyses in present research.

Table 1 shows the sociodemographic distribution of the total, clinical and community sample. 53.3% of the participants of the total sample were women and aged between 18 and 80 years (*M* = 43.12; *SD* = 14.76). The clinical sample had a significant greater proportion of women (64.9% compared to 49.9% on community sample) (*χ*^*2*^ = 21.76; *p* < .001) and lower mean age (*M* = 39.09; *DT* = 14.33 compared to the community sample *M* = 44.32; *DT* = 14.68) (*t* = 21.79; *p* < .001). In the total sample, 1.2% had not completed primary education, 4.5% had completed primary education, 54.4% had completed secondary education and 39.8% had completed university studies. With regard to employment status, 56.2% were working. 36.2% of the sample has been diagnosed with more than one mental disorders. Differences between the community and clinical samples were observed on education level (*χ*^*2*^ = 181.58; *p* < .001) and occupational status (*χ*^*2*^ = 163.53; *p* < .001) (see Table 1). Table 2 shows the diagnoses present in the clinical sample. The most frequent diagnostic categories among patients were Depressive Disorders (38.99%) and Anxiety Disorders (35.53%).

**Table 1:** Sociodemographic variables in the clinical, community and total sample

	Total Sample (N = 1390)	Community Sample (n = 1072)	Clinical Sample (n = 318)
Age *M (SD)*	43.12 (14.76)	44.32 (14.68)	39.09 (14.33)
Women	53.3%	49.9%	64.9%
Not completed primary education	1.2%	0.6%	3.5%
Primary education	4.5%	2.6%	11.0%
Secondary education	54.4%	36.8%	67.3%
University	39.8%	46.3%	18.2%
Being employed	56.2%	62.1%	36.2%

**Table 2:** DSM-5 Diagnoses in patient sample (n = 318)

DSM-5 Diagnoses	*N*	%
Neurodevelopmental Disorders	30	9.43
Schizophrenia Spectrum and Other Psychotic Disorders	22	6.92
Bipolar and Related Disorders	12	3.77
Depressive Disorders	124	38.99
Anxiety Disorders	113	35.53
Obsessive–Compulsive and Related Disorders	15	4.72
Trauma- and Stressor-Related Disorders	77	24.21
Dissociative Disorders	4	1.26
Somatic Symptom and Related Disorders	2	0.63
Feeding and Eating Disorders	6	1.89
Disruptive, Impulse-Control, and Conduct Disorders	6	1.89
Substance-Related and Addictive Disorders	11	3.46
Personality Disorders	29	9.12
Other conditions that may be a focus of clinical attention	2	0.63

### Measures

*Spanish version of the Inventory of Depression and Anxiety Symptoms-II* (IDAS-II; De la Rosa-Cáceres et al., 2020; Sanchez-Garcia et al., 2021; Watson et al., 2012). The IDAS-II is an instrument that assesses the severity of symptoms of depression, anxiety and bipolar disorder during the last two weeks. It is composed of 99 items with a 5-point Likert scale (from 1 = “not at all” to 5 = “extremely”). The items are organized in 18 non overlapping scales (Dysphoria, Lassitude, Insomnia, Suicidality, Appetite Loss, Appetite Gain, Well-Being, Ill Temper, Mania, Euphoria, Panic, Social Anxiety, Claustrophobia, Traumatic Intrusions, Traumatic Avoidance, Checking, Ordering and Cleaning) and an overlapping scale (General Depression). Higher scores are indicative of greater symptom severity for all scales except well-being (higher scores for this scale indicate higher well-being).

In this study, the reliability estimated by Cronbach's alpha coefficient provided values between .71 and .91. These values are similar to previous studies (De la Rosa-Cáceres et al., 2020; Irak & Albayrak, 2020; Watson & O’Hara, 2017; Watson et al., 2012).

*12 items Spanish version of the WHO Disability Assessment Schedule II* (WHODAS 2.0; Vázquez-Barquero et al., 2000; WHO, 2000). This instrument was developed from a set of ICF items to measure functional impairment. Each item is scored on a 5-point Likert scale (from 0 = “none” to 4 = “extreme or cannot do”) which grades the difficulty experienced by the participant in performing a given activity. This instrument provides an overall score ranging from 0 to 100, differentiating between no impairment (0–4 points), mild impairment (5–24), moderate impairment (25–49), severe impairment (50–95), and complete impairment (96–100) according to the ICF classification (WHO, 2013). The estimated reliability through the Cronbach's alpha coefficient was .91 for community sample and .88 for the patient sample.

In addition to the above-mentioned instruments, a questionnaire was administered that included sociodemographic information on sex, age, educational level and employment.

### Procedure

The administration of the instruments in the community sample was carried out through a company specialized in online surveys, accredited with ISO-26362 quality standards (quality standard for the management of online research). Prior to the administration of the instruments, each participant completed a pre-test that assessed his or her reading and comprehension skills and to verified that no automatic responses were made. Before starting test administration, participants were informed of the objectives of the study and were informed of their right to withdraw from participation during the test administration process. After receiving this information, the patients signed the informed consent form. Participants received a reward for their participation in the study consisting of a voucher redeemable for gifts.

Data collection from the patient sample was performed by a psychologist trained in the administration of the instruments. Tests were administered in individual sessions in the mental health centers where they were recruited. Patients received the same prior information as community participants and were informed about the anonymous and voluntary nature of their participation in the study before signing informed consent. They also received a voucher redeemable for gifts.

### Data Analysis

In order to evaluate the ability of IDAS-II scores to discriminate between patients and community samples, means and standard deviations were calculated for the IDAS-II scales and the WHODAS 2.0 total scale score in each sample separately. T-tests for independent samples were used to check the differences between two samples. *Cohen’s d* was applied to calculate effect size. According to Cohen (1992), d-values greater than |0.20|, |0.50|, and |0.80| represent small, medium, and large effect sizes, respectively.

The following formula of Jacobson and Truax (1991) was applied to identify the clinical cutoff that differentiates between the community and patient samples:

$$c=\frac{{ M}_{D}\cdot {SD}_{F}+{M}_{F}\cdot {SD}_{D} }{{SD}_{D}+{SD}_{F}}$$

where M_D_ is the mean of patient group, SD_D_ is standard deviation of patient group (which is expected to be more dysfunctional), M_F_ is the mean of community group, and SD_F_ is the standard deviation of community group (which is expected to be more functional). The 95% of confidence intervals for each cutoff were also estimated. IDAS-II scales values above the clinical cutoff thus indicate more dysfunctional scores for all scales, except for Well-Being, where values above the clinical cutoff indicate more functional scores.

As to assess the ability of IDAS-II scores to explain functional impairment, both samples were grouped to increase variability. Values higher than 25 in WHODAS 2.0 were used to classify persons with moderate impairment. Values higher than 50 in WHODAS 2.0 were used classify persons with severe impairment and ROC analyses were executed to identified cutoffs in IDAS-II scores according to these WHODAS 2.0 defined ICF scores. IDAS-II values with best balance between sensitivity and specificity were used as cutoff, with minimum specificity set at .70 (Power et al., 2013).

Finally, a regression model analysis, was executed to identify scales of IDAS-II which explained total impairment (measured by the WHODAS 2.0). Gender and age were included as controlled variables, as according to previous literature they are relevant variables related with the level of internalizing symptoms (Jalnapurkar et al., 2018; Sánchez-García et al., 2021; Nelson et al., 2018). A stepwise procedure was used to identify the predictive scales.

All analyses were executed using SPSS version 27.0.

## Results

### IDAS-II Clinical Cutoffs to Differentiate Community From Patient Samples

Table 3 shows the means of the IDAS-II scales of the community and patient samples. All scales, except well-being, show higher scores in the patient sample. Effect sizes for all scales show Cohen's *d* values greater than 0.30, except well-being (d = 0.19), and are statistically significant. Medium effect sizes are observed for six scales and large effect sizes for eight scales. The largest effect sizes correspond to the scales of dysphoria (*d* = 1.31), general depression (*d* = 1.22), panic (*d* = 1.18), mania (*d* = 1.14), and traumatic intrusions (*d* = 1.10). Higher total score on patient sample are observed on the WHODAS 2.0 (*d* = 1.24).

**Table 3:** Descriptive statistics among community and patient sample, comparison between groups and clinical cutoff of IDAS-II scales and WHODAS 2.0 total score

	Total Sample (N = 1390)	Community sample (n = 1072)	Patient sample (n = 318)	*t*	*p*	*d*	Cutoff	Confidence interval 95% of cutoff	
	*M (SD)*	*M (SD)*	*M (SD)*					Lower limit	Upper limit
General depression	43.92 (15.33)	39.82 (12.17)	57.73 (16.80)	17.683	< .001	1.22	47.34	46.57	48.11
Dysphoria	21.73 (9.22)	19.18 (7.44)	30.33 (9.46)	19.305	< .001	1.31	24.09	23.64	24.54
Lassitude	12.08 (4.79)	11.13 (4.25)	15.32 (5.10)	13.334	< .001	0.89	13.03	12.78	13.28
Insomnia	13.14 (6.17)	12.02 (5.37)	16.92 (7.14)	11.339	< .001	0.78	14.12	13.79	14.46
Suicidality	7.94 (3.91)	7.27 (2.86)	10.22 (5.71)	8.887	< .001	0.65	8.25	8.02	8.49
Appetite Loss	4.94 (2.59)	4.57 (2.19)	6.20 (3.35)	8.173	< .001	0.58	5.21	5.06	5.36
Appetite Gain	5.81 (2.88)	5.59 (2.69)	6.56 (3.36)	4.711	< .001	0.32	6.03	5.87	6.19
Well-being	21.82 (6.25)	22.41 (5.83)	19.84 (7.19)	5.823	< .001	0.19	21.26	20.91	21.60
Ill temper	9.72 (4.61)	8.91 (3.98)	12.48 (5.47)	10.822	< .001	0.75	10.41	10.16	10.66
Mania	9.58 (4.48)	8.46 (3.69)	13.38 (4.84)	16.759	< .001	1.14	10.58	10.36	10.81
Euphoria	8.17 (3.45)	7.87 (3.20)	9.24 (4.03)	5.568	< .001	0.38	8.47	8.28	8.66
Panic	13.41 (6.89)	11.53 (4.92)	19.77 (8.61)	16.296	< .001	1.18	14.53	14.16	14.89
Social Anxiety	10.44 (5.06)	9.44 (4.11)	13.84 (6.35)	11.642	< .001	0.82	11.17	10.89	11.45
Claustrophobia	8.15 (4.54)	7.35 (3.66)	10.88 (5.96)	10.014	< .001	0.71	8.69	8.43	8.95
Traumatic intrusions	7.06 (4.05)	6.02 (2.99)	10.58 (5.07)	15.297	< .001	1.10	7.71	7.49	7.93
Traumatic avoidance	8.50 (4.06)	7.76 (3.70)	11.01 (4.21)	12.412	< .001	0.82	9.28	9.07	9.49
Checking	5.65 (2.81)	5.19 (2.44)	7.24 (3.36)	10.152	< .001	0.70	6.05	5.90	6.21
Ordering	10.01 (3.97)	9.70 (3.69)	11.07 (4.66)	4.787	< .001	0.33	10.31	10.09	10.53
Cleaning	11.47 (5.05)	11.84 (5.06)	10.20 (4.82)	5.131	< .001	0.33	11.00	10.74	11.26
WHODAS 2.0	19.49 (9.21)	16.87 (6.87)	28.33 (10.28)	18.59	< .001	1.24	21.46	21.00	21.92

*d*: absolute value of Cohen’s d

The cutoff values and their confidence intervals do not overlap with the means of either the community or patient sample. Thus, these cutoff values allow us to reliably differentiate between the two types of samples.

### IDAS-II Estimated Cutoff Point Related to Moderate and Severe Impairment According to ICF

According to WHODAS-2.0, 21.15% of the sample had moderate impairment and 3.53% had severe impairment.

Table 4 shows AUC, cutoffs and sensitivity/specificity values of the IDAS-II scales, using the WHODAS 2.0 values corresponding to moderate impairment (score above 25) and the severe impairment (scores above 50) as criteria. AUC values for most of the scales are adequate, except for five scales that are not able to discriminate between persons without impairment and persons with moderate/severe impairment (i.e. appetite gain, ordering, euphoria, cleaning, and well-being). It is also observed that the sensitivity/specificity shows higher values for detecting severe impairment versus moderate/severe impairment. That is, IDAS-II scores show greater sensitivity and specificity in detecting those with greater impairment.

**Table 4:** Results of the ROC analyses and estimated cut points based on criteria for moderate and severe impairment (N = 1390)

	Moderate impairment (scoring > 25 WHODAS 2.0)				Severe impairment (scoring > 50 WHODAS 2.0)			
IDAS-II Scale	AUC [95%CI]	Estimated cut point	Sensitivity	Specifity	AUC [95%CI]	Estimated cut point	Sensitivity	Specifity
General depression	.872 [.85, .90]	43.5	.864	.705	.946 [.92, .97]	48.5	.959	.706
Dysphoria	.863 [.84, .89]	21.5	.844	.701	.943 [.92, .97]	24.5	.980	.705
Lassitude	.808 [.78, .84]	12.5	.762	.705	.885 [.85, .92]	13.5	.898	.704
Insomnia	.788 [.76, .82]	14.5	.690	.744	.855 [.80, .91]	15.5	.857	.719
Suicidality	.783 [.75, .82]	7.5	.667	.828	.872 [.82, .93]	7.5	.878	.746
Appetite loss	.720 [.69, .76]	5.5	.616	.740	.754 [.68, .83]	6.5	.612	.791
Appetite gain	.657 [.62, .69]	6.5	.503	.739	.610 [.52, .70]	7.5	.388	.776
Well-Being	.312 [.28, .35]	26.5	.119	.714	.204 [.13, .28]	26.5	.061	.743
Ill temper	.762 [.73, .80]	10.5	.667	.761	.794 [.73, .86]	11.5	.755	.742
Mania	.805 [.78, .83]	9.5	.782	.703	.790 [.73, .85]	11.5	.694	.730
Euphoria	.594 [.56, .63]	9.5	.374	.766	.518 [.43, .61]	9.5	.306	.738
Panic	.855 [.83, .88]	12.5	.803	.738	.938 [.92, .96]	14.5	.980	.733
Social anxiety	.812 [.78, .84]	10.5	.759	.734	.876 [.83, .93]	11.5	.837	.702
Claustrophobia	.754 [.72, .79]	7.5	.701	.712	.834 [.77, .90]	8.5	.816	.702
Traumatic intrusions	.831 [.80, .86]	6.5	.793	.720	.915 [.89, .95]	7.5	.959	.702
Traumatic avoidance	.719 [.69, .75]	9.5	.592	.714	.710 [.64, .78]	9.5	.653	.730
Checking	.723 [.69, .76]	6.5	.544	.775	.741 [.67, .82]	6.5	.673	.721
Ordering	.638 [.60, .68]	10.5	.476	.730	.606 [.52, .69]	12.5	.408	.757
Cleaning	.548 [.51, .59]	13.5	.401	.705	.590 [.49, .68]	14.5	.429	.735

### IDAS-II Scales Explaining Impairment

Table 5 shows the results of the regression model of the WHODAS 2.0 scores, adjusting for age and sex. The model is statistically significant, explaining 57.1% of the variance of the WHODAS 2.0 (*F*_12.1377_ = 155.305; *p* < .001). The scales with the highest values of the standardized regression coefficients are general depression (β = .236, *p* = < .001), panic (β = .170, *p* = < .001), claustrophobia (β = .148, *p* = < .001) and lassitude (β = .136, *p* = < .001).

**Table 5:** Linear regression analysis for WHODAS 2.0 score (N = 1390)

	B	SE	*β*	*t*	*p*	95% CI	
						*LL*	*UL*
Constant	-7.019	1.135		-6.186	< .001	-9.245	-4.793
Sex	0.016	0.280	0.001	0.059	.953	-0.533	0.566
Age	0.037	0.010	0.074	3.757	< .001	0.018	0.057
General depression	0.114	0.024	0.236	4.737	< .001	0.067	0.161
Claustrophobia	0.242	0.040	0.148	5.987	< .001	0.162	0.321
Panic	0.183	0.039	0.170	4.658	< .001	0.106	0.260
Social anxiety	0.160	0.042	0.109	3.848	< .001	0.078	0.241
Ill temper	-0.205	0.046	-0.128	-4.451	< .001	-0.296	-0.115
Lassitude	0.210	0.046	0.136	4.539	< .001	0.119	0.300
Traumatic Intrusions	0.180	0.058	0.099	3.089	.002	0.066	0.294
Well-being	-0.097	0.025	-0.082	-3.804	< .001	-0.147	-0.047
Mania	0.169	0.045	0.103	3.784	< .001	0.082	0.257
Cleaning	-0.047	0.021	-0.044	-2.261	.024	-0.088	-0.006

*CI* confidence interval, *LL* lower limit, *UL* upper limit

## Discussion

The current study presents novel evidence on the clinical utility of IDAS-II scores by providing information about the associated degree of impairment. The IDAS-II has demostrated to be a reliable (De la Rosa-Cáceres et al., 2020; Irak & Albayrak, 2020; Sanchez-Garcia et al., 2021; Watson et al., 2012) and useful assessment measure of internalizing symptoms in research using a transdiagnostic approach to emotional disorders (Kotov et al., 2017). Present results complement previous evidence, by bringing forth reliable cutoffs that differentiate patient and community samples and by estimating cutoffs of IDAS-II scales associated with different levels of impairment that can help clinicians with therapeutic planning. Furthermore, our results suggest that General Depression, Lassitude, Panic and Claustrophobia contribute to impairment in a greater extent.

In relation to the first objective, and in congruence with previous studies (Irak & Albayrak, 2020; Watson & O’Hara, 2017; Watson et al., 2012), the patient sample in the current study is more dysfunctional in terms of presence of internalizing symptoms as captured by the IDAS-II scales and higher impairment as measured by the WHODAS 2.0 total score. Most of the IDAS-II scales show moderate to high effect sizes when comparing the community and patient sample, with largest effect sizes for the IDAS-II scales of General Depression and Dysphoria, as hypothesized. These two subscales also best differentiated patients and healthy controls in previous research (Irak & Albayrak, 2020; Stasik-O’Brien et al., 2019). The newly provided cutoff points, that differentiate between patient and non-patient samples with non-overlapping confidence intervals, may allow to use this instrument to detect emotional disorders in primary care patients based of a composite profile, as has been recommended (Ferenchick et al., 2019; Park & Zarate, 2019).

However, it should be noted that the IDAS-II Cleaning scale did not show the ability to provide a reliable cutoff in the current study. This result may be due to the impact of COVID-19 during data collection. A recent systematic review suggested that both patients and healthy individuals have experienced contamination obsessive–compulsive-like symptoms related with COVID-19 (Guzick et al., 2021). Considering that cleaning was a protective factor against COVID-19, and thus ‘healthy’ behavior, it is reasonable that effect sizes on this scale are among the lowest and that the scores observed in the community sample are even higher than those observed in the patient sample. In this sense, it is likely that the assessment of this specific symptom of obsessive–compulsive disorder through the IDAS-II needs to be taken with caution when administered during COVID-19 or similar conditions.

Concerning the second aim, this study provides for the first time cutoffs for different levels of impairment according to the ICF, as measured by WHODAS 2.0. Therefore, present results contribute to the interpretablity of the IDAS-II, assigning qualitative meaning to quantitative scores (Terwee et al., 2007). Up to our knowledge, clinical utility of IDAS-II was limited to the study of its discriminative abitlity to diferentiate between distinct disorders (Stasik-O’Brien et al., 2019). However, emotional disorders generate high disability in different areas of people’s live even beyond patients samples (Guilera et al., 2020). Our study could support this finding, our values of the IDAS-II scales are also differentially associated with moderate and severe impairment in the community sample. Our findings more specifically show that both General Depression and Dysphoria are the scales that most diferentiate among level of impairment. Previous research suggested that the General Depression IDAS-II scale can be used to screen for the presence of internalizing psychopathology (Stasik-O’Brien et al., 2019; Watson et al., 2007). Similarly, Dysphoria has been noted as a nonspecific assessment of core emotional symptoms of depression and anxiety, representing the general distress dimension of psychopathology (Watson et al., 2007). Overall, our findings support to use these scales, for both screening purposes on one hand, as well a to evaluate the impact of internalizing symptoms on impairment on the other hand.

On the other hand, it is necessary to emphasize that some specific scales of the IDAS-II show a limited ability to discriminate between degree of impairment severity in the current study (e.g. euphoria, well-being, traumatic intrusions, checking) and show lower effect sizes. To maximize the proportion of patients correctly classified among the most severe impairment group, the present study applied a .70 as the minimum value for specificity.. Although it might be very interesting to extend the evidence for other sensitivity and specificity parameters in further research, the current results provide useful guidance for clinical decision making (Pintea & Moldovan, 2009).

Finally, IDAS-II scales explained 57% of the variability of the WHODAS 2.0 scores. Consistent with previous studies (Löwe et al., 2008; Tanner et al., 2019), we observed that two of the scales with the greatest explanatory capacity for impairment are Lassitude and General depression. This finding is in congruence with studies that point to depression as the leading cause of disability worldwide and the fact that the disorder is responsible for the highest proportion of disability-adjusted life-years (DALYs) (GBD Mental Disorders Collaborators, 2022; WHO, 2017). In addition, the scales of Claustrophobia, Panic indicate these two anxiety- related symptoms are most related with impairment. Although the relationship between panic and impairment has been widely reported in the literature (Batelaan et al., 2012; Cha et al., 2022), studies associating specific phobias with impairment are scarce and showed mixed results (Burstein et al., 2012; Emmelkamp & Ehring, 2014; Essau et al., 2000; Stinson et al., 2007). In this sense, the present study adds a new piece of evidence to the possible contribution of phobic symptoms (i.e. claustrophobia) to impairment.

From a clinical standpoint, the present study contributes in a novel and useful way to the identification of impairment by providing cutoffs for IDAS-II scales. Despite the benefits that the administration of PROM can bring in clinical routine, several studies indicated scarce use of specific PROM tests (Lewis et al., 2020). For tests and scales to be regularly administered in clinical sessions, it is necessary that their scores contribute clearly to clinical decision making (Kraemer et al., 2004; Widiger & Samuel, 2005). The cutoffs provided in present paper, allow to describe each patient profile in detail and to identify those emotional symptoms on which psychological interventions should target. Moreover, knowledge of which symptoms are most related with impairment, allows healthcare providers to improve treatment planning based on empirical evidence. Finally, the information provided can be useful to assess the impact of treatment by using outcome monitoring. A patient is expected to present with a score above the cutoff at the start of treatment. After several sessions, clinicians can observe whether the patient's score has changed towards the range of functional values (below the cutoff), or whether the observed changes are attributable to measurement error (scores in the range of the cutoff confidence interval).

Despite the promising results, we must also report some limitations. First, the sample of mental health service patients was not drawn by a randomized procedure. Probably, the difference between the patients who participated and those who did not participate lies exclusively in the availability of the time needed to administer the instruments. Therefore, we consider that the impact of this issue on results is limited. Furthermore, although the socio-demographic characteristics of the two samples appeared to be different, these differences are a reflection of different population characteristics, with, for example, the female gender and younger people having higher rates of internalizing disorders in previous research (Jalnapurkar et al, 2018; Sánchez-García et al., 2021).

Second, data from the community sample was collected online, a procedure that, according to Arditte et al. (2016), could lead to higher psychopathological scores compared to other traditional data collection procedures. However, the study by Sanchez-Garcia et al. (2021) using the same data set showed that these do not differ from data from other Spanish samples collected by traditional methods (De la Rosa-Cáceres et al., 2020) nor from the American normative sample used by Nelson et al. (2018). Further, a comparative study by Weigold et al. (2013) indicated equivalence across paper-and-pencil and internet-based data collection methods.

Third, it should be noted that unexpected results have been obtained on scales such as "cleaning". This is probably due to the impact of the COVID-19 pandemic and it is unknown to what extent other scales may be affected.

Finally, we would like to point out that the patient's functional impairment has been assessed using the 12-item version of the WHODAS 2.0 (WHO, 2000). This version has shown psychometric properties equivalent to the 36-item version (Saltychev et al., 2021; Üstün et al., 2010). However, it should be noted that future studies may complement the results of this study with other functional impairment instruments and scales.

## Data Availability

The datasets analysed during the current study are available in Arias Montano, the Institutional Repository of the University of Huelva: http://hdl.handle.net/10272/20953
